# Idiopathic double tracheal stenosis mimicking asthma: a case report

**DOI:** 10.11604/pamj.2023.44.9.36078

**Published:** 2023-01-05

**Authors:** Sarra Maazoui, Tasnim Znegui, Amany Touil, Sonia Habibech, Islam Mejri, Hajer Racil, Nawel Chaouch

**Affiliations:** 1 Department of Pulmonology and Interventional Endoscopy, Pavilion 2, Abderrahman Mami Hospital, Ariana, Tunisia,; 2 Department of Pneumology, Military Hospital of Tunis, Tunis, Tunisia

**Keywords:** Asthma, bronchoscopy, tracheal stenosis, case report

## Abstract

Idiopathic tracheal stenosis (Idio-SS) is an extremely rare disease. Its diagnosis is of exclusion and could be misdiagnosed as asthma. Herein, we report the case of a 39-year-old woman who had been treated for asthma for several months. She has no history of endotracheal intubation or granulomatous disease. Flexible fiberoptic bronchoscopy and thoracic computed tomography revealed double tracheal stenosis. The patient had rigid bronchoscopy; the upper tracheal stenosis was dilated with insertion of a silicone airway stent at the level of the distal stenosis. The diagnosis of idiopathic stenoses was made according to the clinico-radiological features. Symptoms were completely relieved and no recurrence was observed after one year of follow-up. This case highlights the importance of clinical suspicion and early diagnosis of Idio-SS in patients with unexplained wheezing and dyspnea. It also illustrates the role of endoscopic procedures in this situation.

## Introduction

Idiopathic tracheal stenosis (Idio-SS) refers to a narrowing of the trachea of unknown etiology [1]. It is a rare condition that occurred in patients with no history of inflammatory diseases or mechanical injury due to intubation or trauma [2]. It is the differential diagnosis of new-onset severe acute asthma in patients who present to the emergency department with wheezing [3]. We report a case of a 39-year-old Tunisian patient with double idiopathic tracheal stenoses.

## Patient and observation

**Patient information:** a 39-year-old woman with no history of iatrogenic injury complained of symptoms of chest tightness and dyspnea progressively worsening over several months. The patient has never smoked and has no personal or family comorbidities. She was seen by different physicians and treated with inhaled corticosteroids for a presumed diagnosis of new-onset asthma. She presented to us with complaints of progressively worsening shortness of breath and frequent visits to the emergency room.

**Figure 1:**
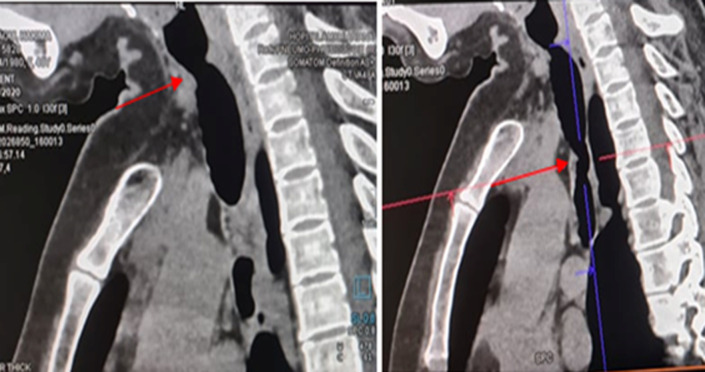
sagittal sections of thoracic computed tomography showing double tracheal narrowing approximately 20 mm below the vocal cords and 40 mm from the carina

**Figure 2:**
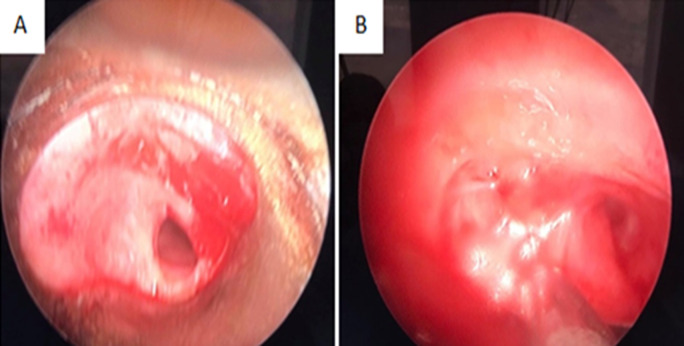
bronchoscopic view of stenosis; A) circumferential irregular cervical stenosis; B) irregular hemi-circumferential tracheal stenosis

**Clinical findings:** the physical examination was marked by stridor on rapidly forced inspiration and quiet expiration. Oxygen saturation was 99%. Chest X-ray revealed normal findings and pulmonary function tests showed fixed upper airway obstruction. Flexible fiberoptic bronchoscopy revealed a short subglottic inflammatory stenosis obstructing approximately 60% of the tracheal lumen, located 10 millimeters of vocal cords, and another distal narrow fibrous stenosis that cannot be catheterized. Thoracic computed tomography revealed two tracheal stenoses (Figure 1). A circumferential irregular cervical stenosis which is approximately 20 millimeters distal to the vocal cords responsible for a 62% reduction. The second stenosis was a distal irregular hemi-circumferential located at 40 millimeters of the carina. It extends over 20 millimeters and is responsible for a reduction of 80% of the tracheal lumen (Figure 2).

**Therapeutic intervention:** thoracic surgery department concluded that surgical intervention was inappropriate, due to diffuse involvement of the trachea. A rigid bronchoscopy was performed; the upper tracheal stenoses were dilated under general anesthesia using various sizes of rigid bronchoscopes (10, 12 then 14) with insertion of a silicone airway stent at the level of the distal stenosis. Several biopsies with lavage were performed during the same operative act. The serological examination was normal anti-nuclear antibody, anti-neutrophil antibody, and angiotensin-converting enzyme levels. The pathology report showed varying degrees of inflammation and fibrosis. No evidence of granulomatous disease or malignancy was present. Therefore, the diagnosis of idiopathic stenosis was made according to the clinical-radiological features and the normality of investigations eliminating infections, malignancy, and systemic diseases.

**Follow-up and outcomes:** after discharge, the patient reported a clinical improvement. Control endoscopy, performed one month later, revealed the subglottic stenosis narrowing about 10% of the airway lumen, and a patent stenting was placed about 60 mm distal to vocal cords. In the respiratory function test, forced vital capacity (FVC) was measured as 3.69 L (95.94%), forced expiratory volume (FEV1) as 2.90 L (92.82%), and FEV1 /FVC as 78.81%. After one year of follow-up, she is feeling better and has no other complaints.

**Consent:** informed consent has been signed by the patient.

## Discussion

Idiopathic tracheal stenosis is a recurrent, rare fibro-inflammatory disease that results in life-threatening blockage of the upper airway. The first cases were described by Brandenburg in 1972. This disease is mainly diagnosed in women aged 30-50 years and results in significant morbidities, such as respiratory distress and intolerance for physical activity [4,5]. Stridor, dyspnea, and chronic cough are the most common symptoms with a mean duration before diagnosis ranging from 19 months to 4 years. These symptoms often lead to a misdiagnosis of asthma or chronic obstructive pulmonary disease and can result in a delay in diagnosis. Other reported symptoms include voice changes and increased mucus production [1]. Diagnosis of Idio-SS is of exclusion. It´s made according to the clinical-radiological features and the normality of investigations eliminating other causes. The known causes of central airway stenosis should be excluded including prolonged intubation, excessive endotracheal tube cuff pressure, tracheostomy, infections (Bacterial tracheitis, tuberculosis, histoplasmosis...), gastroesophageal reflux disorder, radiation therapy, inhalational injury, occupational exposures, tracheal malignancies, and congenital conditions. In addition, upper airway stenosis can be a manifestation of systemic diseases in particular sarcoidosis and its frequency usually increases when parenchymal lung disease progresses [1,3].

Thus, the evaluation should include serologic testing for anti-neutrophil cytoplasmic antibodies (ANCA), angiotensin-converting enzyme levels (ACE), pulmonary function tests, and computed tomography. Bronchoscopy is an important tool, as it confirms the diagnosis and allows characterizing the stenosis, which guides treatment [1]. The principal histologic finding was a fibrous proliferation in the lumina propria of the tracheal mucosa. The cartilage appears intact. The exact pathogenesis of Idio-SS is unknown, but disordered inflammation, aberrant immune response to infection, and gastro-oesophageal reflux are thought to play a crucial role. In spite of a higher female predominance, the hormonal role or the effect of estrogens has not been proven [3,6]. Treatment options range from conservative to definitive surgical procedures. Surgical treatment is invasive and associated with a high level of morbidity. This case was not suitable for surgical treatment because of diffuse airway involvement. The minimally invasive nature of endoscopic techniques and low rate of complications make endoscopic treatment a good first-line treatment for Idio-SS [4,7]. Endoscopic management provides a safe and efficient therapeutic option. Endoscopic techniques include rigid dilation, balloon dilation, laser, electrocoagulation, and (or) stent placement. Stent applications added to dilatation will yield longer palliation in the patient, and it can establish the patient´s comfort without the need for tracheostomy or more invasive processes. Tracheal stenting is reserved for stenoses deemed inoperable for local or general reasons [5,8]. Mechanical dilation and tracheal stenting were successful in restoring tracheal patency.

## Conclusion

Idiopathic tracheal stenosis may mimic symptoms of asthma and has a negative impact on quality of life. This case illustrates the importance of Idio-SS diagnosis in patients with unexplained wheezing and dyspnea, and the role of endoscopic procedures management of this condition.
